# Supplementary material for: Prenatal Risk Factors for Brief Resolved Unexplained Events in Infants
Source: Pediatr Rep. 2025 Feb 6;17(1):16. doi: 10.3390/pediatric17010016 (PMC11858546; doi:10.3390/pediatric17010016)
Supplement: Supplementary file 1 [file pediatrrep-17-00016-s001.zip › pediatrrep-3385741-supplementary.pdf]

On-line Repository

Supplemental Table S1: Medication use during pregnancy

Drugs	Trimester of pregnancy	Controls (n.110), % total (no.)	Cases (n.88), total % (no.)	Chi-square, Contingency tables (p)
Antibiotics	1st	(1)	4.5 (4)	0.123
	2nd	3.6 (4)	5.7 (5)	0.363
	3rd	5.5 (6)	10.2 (9)	0.161
Paracetamol (yes)	1st	17.3 (19)	15.9 (14)	0.476
	2nd	21.8 (24)	23.9 (21)	0.431
	3rd	15.5 (17)	17.0 (15)	0.455
Aspirin (yes)	1st	(2)	5.7 (5)	0.141
	2nd	3.6 (4)	9.1 (8)	0.097
	3rd	1.8 (2)	9.1 (8)	**0.022**
Antidepressants (yes)	1st	(1)	0	0.556
	2nd	-	-	-
	3rd	0	(1)	0.444
Antihypertensive (yes)	1st	-	-	-
	2nd	-	-	-
	3rd	-	-	-
Anxiolytics (yes)	1st	-	-	-
	2nd	-	-	-
	3rd	-	-	-
Anti-inflammatories (yes)	1st	0	(1)	0.444
	2nd	2.7 (3)	(2)	0.604
	3rd	(1)	(1)	0.693
Heparin (yes)	1st	(2)	3.4 (3)	0.396
	2nd	(2)	4.5 (4)	0.243
	3rd	(1)	4.5 (4)	0.123
Thyroxine (yes)	1st	8.2 (9)	8.0 (7)	0.584
	2nd	8.2 (9)	9.1 (8)	0.508
	3rd	8.2 (9)	9.1 (8)	0.508
Insulin (yes)	1st	-	-	-
	2nd	-	-	-
	3rd	-	-	-
Antihistamine (yes)	1st	-	-	-
	2nd	-	-	-
	3rd	0	(1)	0.444

cortisone nose sprsay (yes)	1st	-	-	-
	2nd	0	(1)	0.444
	3rd	0	(1)	0.444
Bronchodilator (yes)	1st	0	(1)	0.444
	2nd	0	(1)	0.444
	3rd	0	(2)	0.196
Antiepileptic (yes)	1st	0	(1)	0.444
	2nd	(1)	(1)	0.693
	3rd	(1)	(1)	0.693

Table S2
Binary logistic regression analysis to predict risk ALTE/BRUE events.

**Continuous variables**	**Categorical variables**	**Variables entered the equation**	**T**	**ES**	**Wald**	**P value**	**Exp(B)**	**95% CI EXP(B)**	
								Inferior	Superior
**Gestational age, Maternal age, BMI before pregnancy, Weight gain during pregnancy**	Hypertension, Diabetes, GER, Cardiac-vascular diseases, Pulmonary diseases, Metabolic diseases.	Gestational age	-0.299	0.101	8,813	0.003	0.742	0.609	0.903
	Snoring in pregnancy (I , I I , III quarters) .	Gestational age	-0.292	0.105	7,759	0.005	0.747	0.609	0.917
		Snoring in 1st trimester pregnancy	-1.636	0.538	9,237	0.002	0.195	0.068	0.559
		Snoring in third trimester pregnancy	1.128	0.434	6,771	0.009	**3,090**	**1,321**	7,226
	Gastroesophageal reflux in pregnancy, Diabetes, Hypertension (I, II, III extreme trim)	Gestational age	-0.332	0.109	9,342	0.002	0.718	0.580	0.888
		GER in pregnancy (III trimester)	-0.858	0.307	7,786	0.005	0.424	0.232	0.775
	Edemas slopes, cramps, moving legs, uterine contractions, nocturia , smoking in pregnancy (I , II, III extreme trim)	Gestational age	-0.307	0.110	7,851	0.005	0.735	0.593	0.912

		Moving the legs II trimester	2,874	1.074	7.162	0.007	**17,710**	**2.158**	145.331
		Uterine contractions II trimester	2,626	1.211	4,704	.030	**13,823**	**1,288**	148,360
	Congestion nasal, Mal Of Throat, Fever, Cough, Pains muscles, Fatigue, Diarrhea (I, II, III extreme quarters) .	Gestational age	- 0.268	0.109	6.033	0.014	0.765	0.618	0.947
		Third trimester muscle pain	2,060	0.731	7,937	0.005	**7,844**	**1,872**	32,874
		Third trimester fatigue	- 1.208	0.390	9,615	0.002	0.299	0.139	0.641
		III trimester diarrhea	- 2,280	1.106	4,250	0.039	0.102	0.012	0.894
	Difficulty falling asleep, difficulty maintain sleep, awakening Early, Satisfaction, Operation daytime, Quality life, Concern (III extreme trim)	Gestational age	- 0.301	0.101	8,872	0.003	0.740	0.607	0.902
	Depression, Loss of interest, Nervousness, Control and worries (last month)	Gestational age	- 0.301	0.101	8,872	0.003	0.740	0.607	0.902

Legend:
T: measures the statistical significance of the relationship between the independent variable and the dependent variable. ES: measures the effect of a one-unit change in the independent variable on the dependent variable. Wald: The Wald test value measures the statistical significance of the relationship between the independent variable and the dependent variable. P-value: represents the probability of obtaining a significant regression coefficient by chance. Exp(B): represents the ratio of probabilities for a given outcome for a one-unit change in the independent variable. 95% CI Exp(B): represents the region with a certain probability (usually 95%) where the true, but unknown exponential of the regression coefficient will be found. If Exp(B) < 1, it means that increasing the independent

variable decreases the likelihood of the dependent variable occurring (protective effect of the independent variable on the dependent variable). If Exp(B) > 1, it means that increasing the independent variable increases the likelihood of the dependent variable occurring (risk associated with the independent variable on the dependent variable).

APPENDIX A
Data collection tools and procedures

Questionnaire 1

Last name name ____________________ mom of_________________________
Gestational age (wks): _______________
Maternal age (years old): ___________
Weight before pregnancy: ____________kg
Weight in pregnancy (full term): _________kg
Height: ________cm

Questionnaire 1A

	Yes	No
Do you suffer from hypertension (high blood pressure)?	☐	☐
Do you suffer from gastroesophageal reflux?	☐	☐
Do you suffer from cardiovascular disease?	☐	☐
Do you suffer from lung diseases?	☐	☐
Do you suffer from metabolic diseases?	☐	☐
Do you suffer from diabetes?	☐	☐

Questionnaire 2A

		FIRST QUARTER	II QUARTER	III QUARTER
Did you snore at night during pregnancy?	Yes			
	No			
	I don't know			
Please answer the following questions only if you answered "Yes" to the previous question				
His snoring was:	a) Slightly louder than a breath			
	b) Loud like talking			
	c) Louder than talking			
	d) Very noisy			
Have you snored often?	a) More or less every night			
	b) 3-4 nights a week			

	c) 3-4 nights a month			
	d) 1-2 nights a month			
Has your snoring ever bothered other people?	a) Yes			
	b) No			
Has anyone noticed that he stopped breathing while sleeping?	a) More or less every night			
	b) 3-4 nights a week			
	c) 1-2 nights a week			
	d) 1-2 nights a month			

Questionnaire 2B

Did you experience any of the following problems during pregnancy? If yes, please indicate which of the following:

	**1st quarter**	**2nd quarter**	**3rd quarter**
Gastroesophageal reflux	☐	☐	☐
gestational diabetes	☐	☐	☐
Hypertension (high blood pressure)	☐	☐	☐
Sloping edema (swollen legs and ankles)	☐	☐	☐
Nocturnal cramps in the lower extremities	☐	☐	☐
Irrepressible feeling of moving your legs	☐	☐	☐
Vigorous nocturnal uterine contractions	☐	☐	☐
Need to urinate several times during the night	☐	☐	☐
Did you smoke during pregnancy? If yes, please indicate in which quarter	☐	☐	☐

Questionnaire 2C

Did you experience any of the following symptoms during pregnancy? If yes, please indicate which of the following:

	FIRST QUARTER	II QUARTER	III QUARTER
Cold and nasal congestion	☐	☐	☐
Sore throat	☐	☐	☐
Fever (>38°C)	☐	☐	☐
Cough	☐	☐	☐

Muscle aches	☐	☐	☐
Tiredness	☐	☐	☐
Diarrhea	☐	☐	☐
Positive COVID-19 swab	☐	☐	☐

2D questionnaire

Did you take a medication during pregnancy? If yes, please indicate which of the following.

Drugs	1st quarter	2nd quarter	3rd quarter
Antibiotics	☐	☐	☐
Paracetamol (Tachypyrine)	☐	☐	☐
Cardioaspirin	☐	☐	☐
Antidepressants	☐	☐	☐
Antihypertensives	☐	☐	☐
Anxiolytics	☐	☐	☐
Anti-inflammatory	☐	☐	☐
CPAP	☐	☐	☐
Specify more:	______________________	______________________	______________________

Questionnaire 3

Insomnia severity index.
Did you experience any of the following symptoms during the third trimester? If yes, please indicate which of the following and its severity:

1. Assess the severity of your insomnia in the third trimester	It is not 0	Mild 1	Moderate 2	Serious 3	Serious 4
Difficulty falling asleep	☐	☐	☐	☐	☐
Difficulty staying asleep	☐	☐	☐	☐	☐
Problem of early awakening	☐	☐	☐	☐	☐
2. How satisfied/dissatisfied were you with your sleep in the third trimester?	A lot dissatisfied	A little	Enough	A lot	Very dissatisfied
	0 ☐	1 ☐	2 ☐	3 ☐	4 ☐
3. How much do you think your sleep problems interfered with your daytime functioning ability in the third trimester? (fatigue , work efficiency, daily chores, concentration, memory, mood, etc.)	Not at all	A little	Enough	A lot	Very much
	0 ☐	1 ☐	2 ☐	3 ☐	4 ☐
4. How much do you think others have noticed your sleep problem in terms of worsening your quality of life in the third trimester?	Not at all	A little	Partly	A lot	Very much
	0 ☐	1 ☐	2 ☐	3 ☐	4 ☐
5. How worried/distressed were you about the problem in the third trimester?	Not worried at all	A little	Partly	A lot	Very much
	0 ☐	1 ☐	2 ☐	3 ☐	4 ☐

Questionnaire 3B

Did you experience any of the following symptoms during the last month of pregnancy? If yes, please indicate which of the following and its severity:

	Yes	No
During the last month have you felt most of the time and days down, depressed or hopeless?	☐	☐
During the last month have you felt most of the time and days little interest or pleasure in doing things?	☐	☐
During the last month have you felt most of the time and days nervous, anxious or restless?	0 never<br>1 some days<br>2 over half days<br>3 every day	☐<br>☐<br>☐<br>☐
During the last month, have you been unable to stop or control your concerns?	0 never<br>1 some days<br>2 over half days<br>3 every day	☐<br>☐<br>☐<br>☐
